# Genomic research and the cancer clinic: uncertainty and expectations in professional accounts

**DOI:** 10.1080/14636778.2019.1586525

**Published:** 2019-03-07

**Authors:** Anne Kerr, Julia Swallow, Choon Key Chekar, Sarah Cunningham-Burley

**Affiliations:** a School of Sociology and Social Policy, University of Leeds, Leeds, UK; b The Usher Institute, University of Edinburgh, Edinburgh, UK

**Keywords:** genomics, cancer, expectations, uncertainty

## Abstract

This paper explores clinicians’ and scientists’ accounts of genomic research in cancer care and the complexities and challenges involved with delivering this work. Contributing to the sociology of (low) expectations, we draw on sociological studies of uncertainty in medicine to explore their accounts of working with uncertainty as part of the management of patient and institutional expectations. We consider their appeals to the importance of modest inquiry and framing of the uncertainties of genomic medicine as normal and at times welcome as they sought to configure professional autonomy and jurisdictions and cultivate an experimental ethos amongst their patients. We argue that these types of uncertainty work [Star, S. L. 1985. “Scientific Work and Uncertainty.” *Social Studies of Science* 15 (3): 391–427] are a key feature of managing expectations at the intersections of genomic research and clinical care.

## Introduction

Genomics is at the forefront of contemporary efforts to develop and deliver personalized medicine. This complex landscape of Whole Genome Sequencing (WGS), molecular profiling, adaptive trials and targeted treatments is associated with a range of transformative healthcare agendas to personalize diagnosis, care and treatment for major diseases like cancer (Beck and Ng 2014). Across the UK, the National Health Service (NHS) and research funders such as Cancer Research UK, working with commercial partners such as Illumina, have invested in a range of research programmes aimed at mainstreaming genomic medicine in cancer care – this includes Genomics England and its flagship 100,000 Genomes Project which aims to, “create a new genomic medicine service for the NHS – transforming the way people are cared for” (Genomics England 2018). The CRUK Stratified Medicine Programme is another “pioneering initiative” which aims to bring the “benefits of genetic testing to lung cancer patients and researchers across the UK” (Cancer Research UK, 2018).

The promise of genomics is, however, tempered by a range of concerns about its transformative potential. Debates about new trial methodologies, measures of efficacy, stewardship and analysis of data, and how to finance genomic medicine can be found across professional literatures (e.g. Conti *et al.*^2010^; Hood and Friend 2011; Faulkner *et al.*^2012^). Concerns about the “genohype” (Kakuk 2006) associated with genomic medicine are also apparent, particularly with respect to the dangers of over-inflated politically-driven promises set against difficulties of implementation in complex and often financially challenged health services like the NHS (Samuel and Farsides 2018) (see also Petersen and Krisjansen 2015; Marcon, Bieber, and Caulfield 2018).

Innovative health technologies have long been associated with ebullient discourses of promise and optimism. The sociology of expectations has documented the ways in which these claims help to advance innovation, recruiting commercial, institutional and patient support and building interdisciplinary agendas (Wainwright *et al.*^2006^; Tutton 2011; Pickersgill 2011; Broer and Pickersgill 2015; Gardner, Samuel, and Williams 2015). This research has also shown that lowered expectations and ambivalence have a role in these processes. Toggling between promissory and pessimistic scenarios maintains innovative momentum in biotechnology (Tutton 2011). And, as Gardner, Samuel, and Williams (2015, 998) have argued, “recalibrating” patient expectations to be “tainted with uncertainty” during this process of enrollment in an innovative alliance.

In this paper, we contribute to this sociology of (low) expectations by exploring how scientists and clinicians account for uncertainty in relation to the expectations of genomic research in the cancer clinic. We investigate where and how uncertainties feature in professional accounts of their efforts to deliver genomic research in cancer care and their engagements with patients, institutions and other professionals in the process. To develop our analysis, we draw from medical sociology and STS literatures on uncertainty (e.g. Fox 1980; Timmermans and Angell 2001; Street 2011), focusing on how the development of “scientific medicine” uncovers and creates uncertainties which have to be managed as part of routine professional practice. As Fox (1980) notes, being a doctor involves cultivating ways of thinking and coping with uncertainty, including a sense of the limits of one’s knowledge and knowledge in the field. Calibrating uncertainties in patient-practitioner interactions is also key to professional autonomy and jurisdictions. New technologies and protocols promising increased certainty can disrupt these processes. For example, as initiatives such as Evidence Based Medicine (EBM) have been taken up in medicine, social scientists have shown how new paradigms based around meta-analysis of biomedical data to generate standardized protocols can sit in tension with more patient-centered rationalities and clinical autonomy (Armstrong 2002), especially in fields with a commitment to “experimental intervention,” e.g. for terminally ill patients (Broom, Adams, and Tovey 2009).

Uncertainty can also be understood as having “generative potential” in biomedical research fields such as autism research (Hollin 2017) and exome sequencing for the genetic causes of disease (Timmermans, Tietbohl, and Skaperdas 2017). Hollin (2017, 225) argues that this kind of “uncertainty work” (Star 1985) makes “research doable and failures comprehensible” thereby maintaining expectations and investment in the possibilities of future advances in the field. Street (2011) argues that uncertainty is a “valuable resource,” creating *opportunities* for new forms of expertise and practice. Similarly, Moreira, May, and Bond (2009) demonstrate that uncertainty is part of collectivizing the work of experts and cultivating “regimes of care” (Moreira 2010) around novel diagnostic technologies for complex life limiting diseases like Alzheimer’s (see also Swallow 2017).

These literatures situate uncertainty as an intrinsic feature of medical and biomedical practice, operating across clinician-patient, professional and institutional boundaries and jurisdictions. This suggests the need for a closer look at how medical and scientific professionals negotiate the uncertainties of genomic research in cancer as part of efforts to manage expectations in the clinic and the institution, not least because of the promise of precision. How do they approach the potential of genomic research to offer more definitive diagnosis and effective treatments for (some kinds of) cancer? What are their experiences and perspectives on the implications of these new forms of knowledge for reaching a more precise diagnosis/treatment nexus and managing patient expectations? What new opportunities and challenges do the uncertainties of genomic research offer for professional practice?

In this paper we explore these questions by drawing on interviews with a range of cancer professionals involved with genomic initiatives as part of their clinical and scientific practice We consider their accounts of the uncertainties involved in genomic research and their consequences in cancer medicine, particularly in relation to interpreting and sharing personalized molecular results with other practitioners and patients.

## Methods

As part of a large multi-sited research project into the implications of cancer genomics for patients, we interviewed 25 practitioners involved with molecular cancer research and diagnosis/treatments for cancer patients in the North of England about their perspectives and experiences of genomic research and its impact on patient care. Interviews provided important insights into how uncertainties are accounted for and understood by practitioners, allowing practitioners a space for critical reflection about their work and its meaning. We note, however, that these accounts offer a partial and incomplete account of experiences and perspectives and do not offer direct insights into how uncertainties unfolded across labs and clinics as part of everyday practice. We are therefore careful to approach these accounts as situated and contingent (Clarke, Friese, and Washburn 2016).

The practitioners we interviewed worked at one of the large teaching hospitals in the region. Fifteen of them were involved with one (in some cases two*) large national genomic research programmes for cancer patients that we followed as part of our research. The first programme was a large-scale sequencing project, focused on service transformation to enable the NHS to provide molecular profiling as standard of care in cancer services. Cancer patients were being recruited for Whole Genome Sequencing of their tumor and healthy cells though no results had been fed back to patients during our research. The second programme was an observational pre-screening study of lung cancer patients, which determined eligibility for entry into a non-randomized multi-treatment arm clinical trial.

The remaining participants (10) were not directly involved in recruiting patients or analysing material as part of these two research programmes but had either engaged with their development and implications as part of their work and/or efforts were being made to involve their departments in one of these programmes. Participants included five scientists (one cytogeneticist, one clinical scientist, one histopathologist, one trainee health scientist (genetics), one clinical bioinformatician), three nurses (one clinical trials assistant, one nurse consultant, one research nurse), two genetic counselors and 15 doctors (one consultant geneticist, six oncologists, four pathologists, one clinician scientist, two physicians and one clinical director for a genomic medicine research programme). As part of their work in the hospital these participants were also involved in a range of other genomic studies, trials and/or molecular testing for cancer (see Table below).

**Table:** 

Large scale sequencing project (5 + 2)	Molecular testing programme and flexible clinical trial (10)	Other (10)
Genetic counselor 1 Genetic counselor 2 Consultant geneticist Nurse consultant Clinical director of regional center Cytogeneticist	Clinical Trials Assistant Research nurse Oncologist 1* Oncologist 4 Oncologist 5 Oncologist 7 Pathologist 1 (local PI) Pathologist 2* Pathologist 4 Consultant Physician 2	Clinician Scientist Oncologist 2 – molecular profiling trials Oncologist 6 – clinical director research facility Trainee Oncologist – special interest in genomics Consultant Physician Pathologist 3 Clinical scientist Histopathologist Trainee health scientist (genetics) Clinical Bioinformatician

We spoke to interviewees about their perspectives and experiences of large-scale genomic research initiatives and developments in cancer care, focussing especially on the implications for patients, services and professional practice. Interviews were semi-structured and lasted between 30 min and 1 h; they were recorded and transcribed verbatim. Ethical approval was granted by the University research ethics committee (reference number: AREA 15-108). Using situational analysis (Clarke, Friese, and Washburn 2016) to explore practitioners’ framing of uncertainties about the meaning and implications of genomic research, we identified three main themes across the transcripts: (i) the complexity of cancer biology (ii) how this complexity relates to handling uncertainty when providing results to referring clinicians (iii) difficult conversations with patients. After introducing interviewees’ perceptions of the challenges of genomic medicine in cancer in the institution in order to set the scene for a more in-depth discussion of uncertainties, we go on to explore each of these themes in turn below.

## Mainstreaming genomic medicine for cancer: challenges and ambivalence

The practitioners we interviewed were working in the context of ongoing efforts to transform cancer services through genomics, with considerable local and national tensions around resources, infrastructures and delivery of ambitious recruitment targets (see also Day *et al.*^2017^). Increasingly expected to work across departmental, disciplinary and institutional boundaries, interviewees were negotiating a range of new demands on their time, resources and expertise. Those working in laboratory or genetic services were also experiencing service transformations as part of one of the programmes we studied, which sought to centralize their activities at a regional level; technological infrastructures (particularly IT) were under strain, and new expectations around the collection, preparation, storage and sharing of tissue and data were also placing additional pressures on their service and how they worked with clinical colleagues. Clinicians working directly with patients were also facing a range of challenges around the level of resources, patient demand and staff shortages. The complexity of the consenting and analysis procedures for these programmes were also placing pressure on their workloads and expertise, and there was a sense amongst many of the people we interviewed that their hospital was in deficit because it was not meeting recruitment expectations for both these studies, as compared with other centers (see also Samuel and Farsides 2018).

Although interviewees expressed considerable enthusiasm and support for genomic research because of the more precise diagnostics and tailored treatments this might afford, years of service reorganization and underfunding have also created a culture of lowered expectations about new initiatives, with several questioning if these initiatives were the “latest fashion accessory” or “pet politician’s project” which would not deliver on its promises. There were considerable doubts about whether targeted treatments would be affordable in practice and about the fairness of resource allocation to expensive medicines for cancer as compared with other priorities, such as the prevention of cancer.

## The uncertainties of cancer and genomics

Genomic research, then, was associated with a sense of inflated expectations about professional and institutional capacities and obligations to deliver service transformation which meant our interviewees were cautious about its potential impact on their practice and patients. In their accounts of wariness around these new initiatives, some professionals highlighted the uncertainties of WGS and its usefulness in the clinic as part of their justification for lowered and lowering expectations, as we now go on to explore.

One way in which this happened was with interviewees querying the usefulness of WGS to identify genomic mutations (as opposed to expression profiling) to delivering targeted treatments, as one oncologist not involved in recruitment for the genomic medicine initiatives in his institution argued:

As a breast cancer doctor, we are lucky in having the two most important targeted therapies in cancer by a million miles – Tamoxifen and Herceptin. Tamoxifen, you do not test for genetic mutations, you check for expression. With Herceptin you do not test for mutations, you test for expression. So that puts me at the start as being in a sceptical position about the role of genetic mutations … And in genomics as it is currently you don’t need whole genome sequencing, you don’t need a Genomics Medicine Centre, you don’t need any of the things in which there is huge commercial interest and government pressure to develop, to look for expression … . (Oncologist 6)

This sense of caution about the clinical utility of WGS was a recurrent theme in interviews. As another oncologist put it: “whilst some of it is close to prime time some of it is actually quite exploratory. But … there’s a danger of selling people a dream that won’t be a reality for them” (Oncologist 1).

This danger of over-promising based on the limited clinical utility of WGS was also articulated in accounts of genomics increasing rather than decreasing uncertainty because it produced too much data too quickly, challenging trial methodologies to establish the effectiveness of therapies. For example, as one oncologist specializing in lung cancer noted,

… the speed is quicker almost than you can actually do the studies to define the role of the new molecular treatments because they tend to be rarer and rarer as you go down the line and then how do you actually do a study when you have … in a centre like this, just ten patients, it’s going to be difficult to do the gold-standard test of any treatment in a randomised control trial. So … the ability to detect new genes is ahead of the ability to test the therapies for those new genes. So, as I say, it’s a bit bewildering. (Oncologist 5)

A similar framing was also apparent in this interview with a genetic counselor working as part of one of the genomic initiatives we studied, who expressed concern about the capacity to report genomic results with certainty to cancer patients in the future:

… genetic counselling is woolly enough as it is, often with risks … we obviously have to tell patients … I’m not going to go in and pretend that we know everything about genetics, I certainly don’t now and I can’t imagine I will do once genomics becomes more routine. I think if anything … the more we discover the more we realise what we don’t know in some ways. (Genetic Counsellor 1)

This sense of proliferating uncertainty was associated with a framing of genomics as revealing of infinite layers of complexity, echoed below in an excerpt from an interview with a pathologist working in a multi-disciplinary service for haematological cancers that has already incorporated genomic analysis into its practice:

… we keep finding more but every time we unravel another layer there’s another layer underneath … I’ve lived through maybe three or four iterations of ‘this is the test that’s going to make everything else redundant’ … next generation sequencing is now the next great white hope that’s going to be giving us all the information that we need but … tumours are amazing things … we’ve just talked about resistance … so they may well keep developing resistance and it may be involving things other than the specific genetic mutations … we’ll constantly be running, chasing to catch up. We may eventually get there. (Pathologist 3)

Here uncertainty was presented as endemic to the pursuit of scientific knowledge and the need for caution about the “latest” revolutionary technology was invoked.

Querying the promise of precision and the prospects of service transformation, these professionals performed a medico-scientific ethos of persistent but modest inquiry redolent of the “acceptance of medical uncertainty” and the “positive philosophy-of-doubting” (Fox 1980, 7) described in Fox’s landmark study of Ward F-Second (the metabolic research ward of the Peter Bent Brigham Hospital in Boston) ([1959] 1974). Their accounts, from different clinical and laboratory positions, mobilized uncertainty as a reason to lower expectations around genomic medicine, but not to give up on it entirely – “We may eventually get there.” Reflecting on their experiences with various cancers, treatments, trials, results and tests, practitioners framed uncertainty as endemic, necessitating scepticism about promises of precise diagnosis and effective targeted treatments.

We can also identify a similar narrative in the accounts of nurses involved with these genomic research initiatives, echoing the point above by the pathologist about uncertainties arising because of the sheer complexity of cancer tumors. Nurses negotiating difficult and complex consent and tissue collection procedures spoke about genomic research being one part of “trying to keep one step ahead” of cancer (Research Nurse 1), situating research as part of a wider process of managing a disease marked by uncertainty, evolution and mutation. This was elaborated by one nurse consultant who was part of the leadership team for one of the initiatives we studied:

… just understanding that the gene does this and therefore we can block it with this … would be lovely if that was the end of the story but of course genes mutate and you can have a mixed response within cancer as well … we also know that when we stop some of these drugs you can get a rebound of their cells growing again … So this has been a whole new area of research over the last … years … it isn’t going to come to a happy ending all of a sudden, it will form the basis of ongoing work that will extend people’s lives … we hope for cure but where we can’t cure we hope to make it chronic and manageable and this is one part of that armament really … . (Nurse Consultant 1)

Although the language of “armament” deploys a familiar war-based metaphor concerning cancer (Sporn 1996), this was presented as a long war of attrition, framing cancer as a resistant foe and genomic research as another staging post along the way.

Interviewees also described how uncertainties could arise because of difficulties with delivering complex genomic research protocols and other competing requirements to provide care to patients: tissue sampling was a particular issue here. Samples were sometimes difficult to obtain, especially if the patient was very unwell or their tumor was difficult to reach. Samples could also be very small and be required for multiple (not just genomics) tests, becoming exhausted in the process. But they were also important passports to novel targeted treatments which might be life-saving, as well as important for the numerous research studies seeking to pool and analyse genomic data to develop better diagnostics and treatments for patients in the future. Interviewees told us their experiences of navigating the practicalities of biopsy procedures and analysis could generate uncertain results as in this excerpt from an interview with a pathologist:

It’s not the case that you’ve had a biopsy and you put it through a machine and you get an answer that says, ‘yes you will or will not respond to a particular drug’. Often there’s insufficient material for testing, that means going back to rebiopsy patients which is often inconvenient and potentially associated with risks and it doesn’t necessarily give you a black and white answer. (Pathologist 2)

These accounts were common across our interviews with pathologists (and nursing staff), particularly those involved in the two studies we followed as sample failure had been an issue in both cases. This led them to downplay expectations of tissue analysis, rendering it a provisional and precarious process.

Across these accounts, genomic research initiatives were situated as part of a process of making cancer manageable, with practitioners acknowledging and working with uncertainties as a way of keeping expectations of patients and project leaders in check. In so doing, the interviewees sought to avoid the pitfall of overpromising but at the same time signaled a commitment to these initiatives as part of routine professional practice and the evolution of knowledge.

## Interpretation, uncertainty and professional jurisdictions

We turn now to look more closely at how professionals spoke about the uncertainties involved in interpreting genomic results with and for other professionals, focusing on how their accounts sought to manage expectations about changing professional roles and responsibilities, particularly for pathologists and geneticists. Echoing discourses about the deluge or slew of genomic data which can be found in professional literatures (Prainsack 2017), these respondents expressed concerns about how to interpret large quantities of genomic data and proceed to treatments in the short-term because of a lack of guidelines on interpretation. So, whilst these professionals accepted and foregrounded the uncertainties of cancer genomics as a reason to lower expectations, as described in the previous section, they also expressed a range of frustrations about how to work effectively in the absence of certainty in the context of criticism and threats to their autonomy and jurisdictions. As Fox writes:

The development of scientific medicine, then, has both uncovered and created uncertainties and risks that were not previously known or experienced. Some of these problems are so new, and raise such intricate and important questions of fact, technique, judgment, authority, and values, that they cannot be quickly or neatly resolved. The indeterminateness of these perplexing issues has contributed to the sense of uncertainty about uncertainty, and augmented the sense of risk about risk. (1980, 19)

For professionals involved in interpreting genomic data these uncertainties about uncertainties were expressed in terms of frustrations centered around a lack of literature and guidelines for analysis, as captured below in the excerpt from an interview with a consultant geneticist involved in the large-scale genomics medicine initiative we studied:

In the short-term we don’t know what to do with the data … So … you do a genome sequence on someone, there’s thousands and thousands of variants there. There’s not enough evidence to know what to do with most of the results. … there aren’t the downstream guidelines on what to do with the results. (Consultant Geneticist)

Concerns about a lack of evidence or literature as a basis for interpretation were typically entangled with concerns about professional jurisdictions. These were seen as being encroached or devalued in genomic research initiatives, the goal of which is to mainstream the interpretation and delivery of genomic results. For example, pathologists expressed concerns that the sheer volume of genomic data defied interpretation using standard, “human,” means, as in the excerpt below:

… one concern which is not so much for me but if I was starting off was that I’d actually be made redundant by all of this (laughs) … because you will get certain people that preach that we don’t need any interpretive [skills] you just chuck it in a black box and get a result and that’s all you need. So it removes … the human … . I hate to cast aspersions but you see a lot of genetics laboratories, they must have the best kit, the new test … they’re very good at doing the test but they have no idea about the context … the beauty of a place like this is it considers all the elements and puts it in a clinical context rather than just hiving it off to a laboratory anywhere … [we need to] make sure it evolves the right way and it’s still embedded in a clinical service … because without that interaction the development will grind to a halt because you won’t have input from different perspectives, which is important. (Pathologist 3)

For this pathologist there was a danger that uncertainties around how to interpret results could be resolved or side-stepped by automation or analysis-at-a-distance from the clinic, creating a sense of “displacement” (Bourret, Keating, and Cambrosio 2011) and uncertainty about the decline of the profession and their working arrangements with other clinicians. This elicited an account of the importance of pathology services best embedded in a clinical environment to produce better kinds of interpretation of results: akin to the bio-clinical collective described by Bourret, Keating, and Cambrosio (2011). The importance of pathologists with a keen scepticism about the limits of clinical interpretation working alongside genetics and clinical scientists to “provide clinical oversight about the appropriateness of some of the clinical interpretations” was also stressed:

… other centres will offer blanket clinical interpretations which may not necessarily be appropriate and the problem is oncologists act on those clinical interpretations [especially] if you’re a busy general oncologist working in a smaller hospital you’re not necessarily up to speed with all the evidence … then you may take at face value the clinical interpretation attached to these reports. (Pathologist 2)

Here the capacity of professional groups such as pathologists and clinical geneticists to deliver genomic medicine was asserted on the basis of their expertise in recognizing and managing uncertainty, including their ability to identify the need for appropriate kinds of support in the form of guidelines, literature and multidisciplinary working to mediate that uncertainty. Through these kinds of accounts practitioners with a track record of interpreting clinical data and delivering diagnoses sought to rework the expectations of genomics transforming services in such a way that they could play a key part in its delivery. As Latimer *et al.* (2006, 599) noted, “moments of ambiguity and deferral create an imperative space that helps legitimate the need for more technoscience, and consequently, more clinical judgment with which to fix the genetic future.”

Geneticists also spoke to us about the need to recalibrate the expectations of treating physicians who wanted more certainty from genomic results than they could provide as captured in the excerpt below:

So we quite often send reports out saying, well we’ve seen this … at the moment there’s no literature available to indicate that it’s pathogenic … and that’s our best guess to evidence that. … And for us we think we’ve done as much as we can and the clinician phones: ‘what does this mean?’. (Cytogeneticist)

As in Timmermans’ (2015)’ study of exome sequencing and Variants of Unknown Significance, managing uncertainties around matching genotype and phenotype was exacting work (Timmermans, Tietbohl, and Skaperdas 2017). In the words of another pathologist, reports are “not always as tidy as is sometimes portrayed in the media,” and they can be disappointing, when, for example, results only provide information about patients who are unlikely to respond to particular treatments rather than the ones that are likely to respond – offering “negative predictors, not positive solutions.” Here pathologists and geneticists articulated their key role in mediating uncertainties in pressurized working environments which sometimes resulted in tension with other clinicians’ expectations.

The difficulties of meeting clinicians’ expectations of support with interpreting genomic results in conditions of interpretive uncertainty was also highlighted in relation to new pressures on workload, as in the excerpt below:

… there’s an increasing tendency for other labs and research groups to have access to genetic testing … I got an email the other day from a Haematologist saying, ‘I’ve got a patient. I think they’ve got this condition. It’s all a bit odd. They’ve had some testing through some sort of semi-research study. They’ve found something, here’s the report, what do you think?’ And it becomes clear that actually the people who’ve done the test don’t fully understand what they’ve done … or what to do with the results (laughs). … so you know we’re kind of picking up the pieces in Genetics … which creates more work, and of course that work is not funded … [it gets even more difficult] when someone might have access to a whole exome or genome panel and they’ve not actually worked in a genetics service and the probability of them finding something and they don’t know what to do with it, it just goes up … they’re going to find loads of things. And they’re going to go, ‘Oh, hang on, phone a friend!’. (Consultant Geneticist)

This excerpt illustrates the difficulties this geneticist was navigating with respect to his service and professional role in the context of genomic data – here this respondent flags the ad hoc kinds of research and consultations that are being requested as genomics enters the clinic, asserting his epistemic authority in the context of challenges posed to their service by developing expertise elsewhere. He also flags the difficulties of providing specialist input whilst experiencing budget constraints, further emphasizing concerns about the devaluing of expertise alongside the challenges to professional jurisdiction that genomics might pose.

In this section, we have explored a range of professional accounts of the challenges and difficulties of interpreting and providing genomic data, including for other clinicians. Accounts stressed the complexities and provisional aspects of analysis and emphasized the importance of appropriately resourced, integrated patient-centered services as a way to manage uncertainties appropriately, and as a counter to the threat of automation, de-professionalisation and outsourcing. Echoing the findings of Timmermans (2015; Timmermans, Tietbohl, and Skaperdas 2017), Bourret, Keating, and Cambrosio (2011) and Haase, Michie, and Skinner (2015), professionals gave accounts of evolving local forms of best practice, in this case premised on integrated and bespoke services, carving out a kind of “local universality” (Timmermans and Angell 2001) in the face of concerns about de-skilling and centralization.

## Difficult conversations with patients

In this section, we turn to look more closely at how doctors and nurses accounted for uncertainties around genomic research and data when interacting with patients.

We found that oncologists concurred with pathologists’ and geneticists’ accounts of clinicians finding uncertainty problematic as outlined above, as in the excerpt below from one of the leading proponents of genomic research in the institution:

… so you get into a situation where you’ve actually got to know something about the constellation of problems that the patient had got in order to know which is the particular drug that’s effective. … you could easily imagine the situation where it becomes too complicated for your everyday oncologist to be able to handle it … [or] where its actually too complicated for a human being to understand it … my top line vision is that this is coming, I wouldn’t get into this kind of conversation with anybody other than somebody who really got it about genomics. Because I think it’s frightening … all this complexity is actually frightening to clinicians who love order and control (laugh). And to patients as well of course, who want some control over their destiny as well. (Oncologist 1)

In addition to invoking the threat of automation, this oncologist notes that genomic complexity could amplify unwelcome uncertainties and noted how unsettling this could be for “everyday” practitioners and patients, framing genomic appreciation and understanding as a kind of elite forward-looking activity. Another senior interviewee, tasked with leading a major genomic sequencing initiative in the region, also framed (other) clinicians’ reservations about genomic information in the clinic as a feature of an established “medical culture”:

a significant group of people see certainty in information, it gives much more precise details of what’s going to happen to a patient, is actually something they’d rather not know … it’s easier if you’ve got a 95% chance of being cured, but if you say to someone ‘actually we know from this data that this is just not going to work’ then that’s something people reject. Because if you open the box then you get the information. If you don’t do it in the first place you just carry on regardless. … people actually see personalised medicine as something potentially very, very difficult … particularly what you do when the data shows that somebody really isn’t going to recover and there’s nothing you can do for them … people … [are] quite prepared to crack on with treatment … just for the sake of doing something … . (Clinical Director 1)

Here opening the black box (using genomic technologies that the first of our interviewees above describes as “a bit science fiction”) could generate unwelcome kinds of certainty about treatments not being effective, and in contrast to the previous account, shutting down the possibility of “just carrying on regardless.”

The difficulty of navigating the amplification of unwelcome un/certainties for patients resulting from genomic data was a recurring theme across our interviews with clinicians. Handling the uncertainties of results, treatment decisions and further options in dialogue with patients and families is a major part of cancer practitioners’ work, often involving lengthy complex and sometimes highly technical discussions, together with a keen sense of what kinds of information and choices are appropriate for sharing at what points in the process. Genomics was framed as bringing added complexity to these tasks and accounts of how clinicians would have to carefully navigate this as part of their research and care (see also Latimer *et al.*^2006^; Haase, Michie, and Skinner 2015). As we now go on to explore, the cancer clinicians we interviewed advocated patient-centered management of uncertainty to manage expectations and protect the integrity of their clinical judgement and expertise (Broom, Adams, and Tovey 2009).

The commitment to patient-centered management of genomic uncertainty emerged in a range of accounts, often focused on how best to deliver results to patients. As one clinician scientist with expertise in skin cancer, where genomic analysis is already an established as part of treatment-decision making, commented:

It’s possible to really distress patients by forcing data on them that they really don’t want. You know, some people just do not want to know a percentage, they don’t want it. … it’s my belief anyway that you have to find out how much information your patient wants and needs and give them every access to it … But everyone’s different. (Clinician Scientist)

Too much information, be that survival statistics or genomic data, was presented as a recipe for confusion and anxiety. But for other patients, practitioners argued that too little information could cause anxiety. This is echoed in the excerpt below, where an oncologist specializing in gynaecological cancers involved in a number of trials of molecular analysis, framed genomics as presenting clinicians with the challenge of working out what information to share with patients:

… I think the patients have been amazingly receptive and understanding … you know, ‘We’ve had a look at 592 genes and proteins and these are the genes that have got the abnormalities and this means this and this means that’ is a conversation they’ve largely been capable of listening to with interest. … [But] some patients [say] ‘Well that’s all detailed nonsense, I don’t really understand. What do you think I should do next?’ and they’re quite happy for me to do all the interpretation … The real challenge is working out how much the patient wants to know and then giving them the right information that’s right for them because the right information for one patient is not the right information for the next one. (Oncologist 2)

This need to manage expectations through tailored provision of more detailed layers of information, is a form of personalization with which cancer clinicians are already very well versed. But in these accounts, they spoke of how genomics intensified these processes, and brought with it the need to develop new ways of simplifying information, as this oncologist went on to describe:

… I don’t think it necessarily means more face-time … We’ve moved beyond the days of ‘You’ve got x cancer and therefore you need to have this drug’. I would say, ‘We’ve looked in detail and for your particular type of cancer then we think the right thing to do is this’. Now if somebody said to me, ‘What do you mean, my type of cancer?’ then I would be happy to explain that. But I don’t actually go through … ’We used to think it was x cancer now we recognise its three things, actually those three things could probably be 20 things and those 20 things have now got 1000 individual genetic mutations, so let me take you through the 20,000 possibilities of what it could be … ’ I would just say, the information we have about your cancer means this is the right thing for you … that’s what the patient wants to hear, ‘we know more and more about you which enables us to focus our treatment more and more on you as an individual’. (Oncologist 2)

In this excerpt, although the oncologist begins by saying these developments will not necessarily lengthen consultations, the account suggests that, when patients do ask questions, further efforts to tailor information are necessary to provide reassurance.

Genomic research was also framed as generating new kinds of clinical work in handling disappointments and misunderstandings about the potential for cure which the discourse of precision can generate, particularly since experimental trials can fail as a research nurse involved in a clinical trial in lung cancer explained:

… one of the things we’re having to talk about … is we will understand some of the information we get and we don’t understand some of the other information we get. … I think a lot of our talk now is about how this is going to enhance care, but what about the people who get no useful information back, or the testing fails? (Research Nurse)

Practitioners spoke about having to carefully navigate what kinds of un/certainties participation in research, including trials, could bring to patients, which at times meant downplaying the prospects of certainty and maintaining the possibility of uncertainty and failure given their experimental nature. For example, an oncologist spoke about the need to downplay patients’ expectations of immediate success whilst also cultivating hopefulness about the option of future molecular treatments on trial arms. This came up in discussions about the initial consultation about involvement in the trial:

So we’ll go through about 4 or 5 scenarios and by that stage they’re absolutely saturated [with information] … so we will mention the genomic medicine trial, ‘we’re doing this study which doesn’t involve anything else apart from blood tests. It’s looking for a potential treatment. You may or may not be eligible for it’. So, it’s a sort of loose conversation … it’s not a focus because it’s not a first line treatment. (Oncologist 4)

Concerns about managing patient expectations also arose in relation to handling results that might show that some of the latest treatments would not be effective on a patient’s cancer, as an oncologist with expertise in genomic analysis in the treatment of lung and breast cancer patients commented:

they’d been given a hope … , that science would come to the rescue. You know, and not just a … vague hope, but a very kind of targeted precise hope … that genomics was going … to save the day for them. … that’s doubly difficult for patients … to know that genomics isn’t going to save the day and that there’s no treatment for you and that your life expectancy by necessity … will be shortened. (Oncologist 1)

Here genomic results to do not deliver a targeted treatment are presented as having the potential to make things “doubly difficult” because of the disappointments that can accrue when expectations are not met.

In these discussions, practitioners foregrounded the work involved in navigating the complexity and uncertainties of genomics when caring for patients and managing their expectations. Practitioners told us about how genomic research intensified the complex work involved in navigating how much of what kind of information and opportunities to share with patients at what points in their treatment and for what kinds of patients. Lowering expectations (Gardner, Samuel, and Williams 2015) and “tinging” their encounters with vulnerable patients with uncertainty (Moreira 2010) about the prospects of genomic research were part of their practices of care.

## Discussion and conclusion

Genomics research is increasingly embedded in cancer care. In this article, we have explored how professionals account for uncertainty in the course of managing expectations about what this research can deliver for patients and services more generally. Our analysis has revealed three interconnected kinds of “uncertainty work” (Star 1985) involved in cancer professionals’ encounters with genomic research. First, we demonstrated how reflection on the un/certainties revealed in genomic research about cancer, are part of their articulations of an ethos of modest, persistent inquiry which is linked to their cautious support for genomic research and innovation which also seeks to lower expectations about what this new field can deliver in clinical care. We also explored “uncertainty about uncertainty” in relation to interpretation of genomic results. Here we focused in particular on pathologists’ and geneticists’ concerns about the implications of genomics for their autonomy and jurisdictions, via the articulation of their role in bioclinical collectives (Bourret, Keating, and Cambrosio 2011) that integrated patient-centered care and their important role in resolving clinical uncertainties. In so doing practitioners were engaged in recalibrating other professionals’ and project leaders’ expectations about what can be delivered by genomic research and securing their place in the genomic medicine of the future. In the final section we considered how the uncertainties of genomic research intensified clinicians’ work of managing patients’ expectations through tailoring and personalizing their treatments and research involvement. We discussed how this involved the cultivation of an experimental ethos amongst patients, and the careful navigation of un/welcome kinds of un/certainty generated by too much and too little genomic information arising from their participation in trials.

Through this study we have shown, as genomic research and care develop in the cancer clinic, professionals articulate a range of uncertainties as part of their efforts to recalibrate patient, practitioner and institutional expectations of its prospects. In so doing professionals like pathologists and clinical geneticists are crafting a place for themselves in genomic medicine research and the services of the future, based on their extensive capabilities and experience in navigating other professionals’ expectations of genomics. Oncologists and nurses are also articulating a role for themselves based on their expertise in patient-centered tailoring of genomic information as part of managing patient expectations. Together these practitioners also appeal to the intrinsic uncertainties of cancer as a means by which to moderate expectations of genomic research and innovation at the same time as supporting these initiatives as part of their commitment to scientific progress. As such these types of professional uncertainty work are a key feature of managing expectations at the intersections of genomic research and clinical care.
